# The role of age, sex, and multimorbidity in 7-year change in prevalence of limitations in adults 60–94 years

**DOI:** 10.1038/s41598-022-23053-8

**Published:** 2022-10-31

**Authors:** Benjamin Landré, Andres Gil-Salcedo, Louis Jacob, Alexis Schnitzler, Aline Dugravot, Séverine Sabia, Archana Singh-Manoux

**Affiliations:** 1 Université Paris Cité, Inserm U1153, Epidemiology of Ageing and Neurodegenerative Diseases, 10 Avenue de Verdun, 75010 Paris, France; 2 Research and Development Unit, Parc Sanitari Sant Joan de Déu, CIBERSAM, Sant Boi de Llobregat, Barcelona, Spain; 3 Faculty of Medicine, University of Versailles Saint-Quentin-en-Yvelines, Montigny-le-Bretonneux, France; 4 Department of Epidemiology and Public Health, University College London, London, UK

**Keywords:** Geriatrics, Epidemiology

## Abstract

Recent data suggest a temporal trend in decline in functional limitations in older adults but whether this trend extends to the period after the 8th decade of life remains unclear. We examined change in prevalence of limitations in activities and instrumental activities of daily living (ADL and IADL) between 2008 and 2015 among adults of 60–94 years and the role of age, sex, multimorbidity; we also examined changes in severity of limitations. Data were drawn from two nationally representative surveys in 2008 (n = 13,593) and 2015 (n = 13,267). The 6-item scales of ADL and IADL were each categorized first as ≥ 1 limitations, and then to examine severity as 0, 1–2, and ≥ 3 limitations. Weighted logistic and multinomial regressions were used to estimate prevalence of limitations; the difference between surveys were extracted every 5 years between 60 and 90 years. The prevalence of ≥ 1 ADL declined between 2008 and 2015, from age 75 (− 1.2%; 95%CI = − 2.0, − 0.4%) to age 90 (− 8.8%; 95%CI = − 12.7, − 5.0%). This decline was more pronounced in men than women (p-value for interaction = 0.05) and observed primarily in those with multimorbidity (p-value for interaction = 0.06). Up to 2 ADL limitations declined from age 75 (− 1.0; 95%CI = − 1.7, − 0.3) to 90 (− 6.7; 95%CI = − 9.9, − 3.6) and from age 80 (− 0.6; 95%CI = − 1.1, 0.1) to 85 (− 1.2; 95%CI = − 2.2, − 0.1) for ≥ 3 ADL limitations. There was no substantial change in IADL limitations. These data from a high-income country with universal health care show improvement in ADL even after the 8th decade of life despite increase in multimorbidity.

## Introduction

The proportion of adults aged 60 years and older is increasing globally; it stands at 12% in the global population and is projected to double by 2050, with a more pronounced increase in high-income countries^1,2^. The ageing of populations presents health and social care challenges due to increase in chronic diseases, multimorbidity, and disability at older ages^3,4^. Ageing is characterized by an increase in functional limitations with age^2,5,6^, commonly assessed using Activities of Daily Living (ADLs) and Instrumental Activities of Daily Living (IADL)^5,7^ which correspond to basic and cognitively challenging activities of daily life, respectively^8^. In Europe, between 15 and 35% of persons over 75 years report ADL or IADL limitations^2^.

In high-income countries there is emerging evidence of a decline in the prevalence of functional limitations in adults aged 60 years and older in recent years with heterogeneity across countries^5,9–13^. This decline is more pronounced in ADL than IADL in older adults up to 80 years in data from the Survey of Health, Ageing and Retirement in Europe (SHARE)^5^. Inconsistent results have been observed regarding sex-related trends in limitations with some studies showing greater improvement in functional limitations in women^14,15^ and others similar trends in men and women^16,17^.

There remain four outstanding issues regarding the temporal trend in functional limitations. One, the number of adults in the 75+ age-group is growing faster than any other age-group in high-income countries^18^, and the extent to which functional limitations have also declined in this group remains unclear^19^. Two, how narrowing sex differences in life expectancy over the last decades^20^, including in France^21^, translates into change in functional limitations at older ages in men and women needs to be elucidated. Three, the clustering of chronic diseases, multimorbidity, is common at older ages^3,22,23^ and the manner in which it affects change in functional limitations merits further examination^7,19^. Chronic diseases are known to be important determinants of functional limitations^24–26^, and it is unclear if improvement in functional limitations is evident in those with multimorbidity. Better management of some chronic conditions may well have attenuated their impact on functional status whether this is also the case in the oldest age-groups remains unknown. Four, whether the temporal pattern is similar when severity of functional limitations is considered remains under-investigated^13,27^.

Accordingly, our objective was to compare ADL and IADL limitations using two nationally representative surveys from 2008 (*Handicap-Santé*), and 2015 (*Capacities, Aids and REssources*) in adults aged 60 to 94 years to examine the role of age, sex, multimorbidity, and severity of ADL and IADL limitations.

## Methods

### Study population

Data are drawn from two national cross-sectional surveys undertaken in 2008 and 2015, Handicap-Santé^28^ (HS-2008) and Capacities, Aids and REssources^29^ (CARE-2015), by the French National Institute of Statistics and Economic Studies (Institut National de la Statistique et des Etudes Economiques, INSEE) and the French Directorate of Research, Studies, Assessment, and Statistics (Direction de la Recherche, des Etudes, de l’Evaluation et des Statistiques, DREES). The surveys contribute to the European level effort to collect data on health and disability, coordinated by Eurostat, the statistical office of the European Union. The measures included in the surveys are standardized across the participating countries. The surveys used in our analyses include people living in the community and in institutions (nursing homes, assisted living facilities, and retirement homes). Further details on the methodology used in the surveys are available elsewhere^30^. Briefly, in France the survey design was piloted in a preliminary survey, Vie Quotidienne et Santé (VQS), undertaken by INSEE and DREES. These data were used to set up the sampling framework in HS-2008 and CARE-2015, where the goal was to over-sample people with disability and lead to nationally representative data using weights. Both HS-2008 and CARE-2015 used face-to-face computer-assisted interview undertaken by trained investigators. Our analyses consist of secondary analyses of data collected for surveillance of the health and disability status of the French population; in the present study our focus is on adults aged 60–94 years.

### ADL and IADL limitations

The surveys used French versions of the Katz ADL^31^ and Lawton IADL^32^ scales comprised of 6 ADLs (bathing, dressing, toileting, transferring, feeding and continence) and 6 IADLs (using the telephone, shopping, housekeeping, managing medication, using transport, handling administrative procedures) respectively. Participants reporting major limitations (“Yes, a lot of difficulties”) or inability to carry out these activities without help (“You cannot do it on your own”) were considered to have the limitation in question.

The sum of the number of limitations yielded an ADL and IADL score, each ranging from zero to six. These scores were categorized in two ways, first in the conventional manner by dichotomizing them as zero versus one or more limitations, and then to examine the role of severity of limitations as zero, one to two, and three or more limitations.

### Multimorbidity

Multimorbidity was defined as the co-occurrence of two or more chronic diseases out of a list of eight conditions: stroke, heart disease, diabetes, cancer, musculoskeletal diseases, arthritis, neurodegenerative diseases, and depression. During the face-to-face interview, participants were asked to report all their doctor-diagnosed chronic conditions and the list of 8 conditions was chosen to represent the most common conditions.

### Covariates

Socio-demographic variables were age, sex, and education (none, primary education, high school, baccalaureate, and university or higher degree).

### Statistical analysis

The analyses were undertaken on two outcomes: ADL and IADL using the same procedure. All analyses were weighted to reflect the French population using weights provided by INSEE, using the sampling weight procedure (pweight) from the survey [SVY] commands in Stata. All models included age terms (age, age^2^), survey (HS-2008, CARE-2015), survey by age interaction, sex, multimorbidity, and education. Higher order interactions with age were also examined for each of these covariates and retained in the model when significant on the basis of the Wald test (α = 0.05).

First, the temporal trend in limitations was examined as a function of age. Weighted logistic regression was used to examine differences in prevalence of limitations between surveys along with 95% confidence intervals at age 60, 65, 70, 75, 80, 85, and 90. We assessed whether the temporal trend in limitations differed as a function of age based on the interaction term between survey and age terms (age alone or age and age^2^ depending on the terms retained in the model) using a Wald test.

Second, we examined whether the temporal trend in limitations differed in men and women by adding interaction terms between survey and sex and between survey, sex, and age to previous models. Weighted logistic regression stratified by sex was then used to estimate differences in limitations between survey separately for men and women, with estimates extracted at age 60, 65, 70, 75, 80, 85, and 90 years.

Third, we assessed whether the temporal trend in limitations was similar among individuals with and without multimorbidity using interaction terms between survey and multimorbidity and between survey, multimorbidity and age. Weighted logistic regression stratified by multimorbidity status was then used to estimate difference in limitations between the surveys, separately in participants with and without multimorbidity, with estimates extracted at age 60, 65, 70, 75, 80, 85, and 90 years.

Fourth, the severity of functional limitations was considered by recategorizing the 6-point score as zero, one to two, and ≥ 3 limitations. The difference between the two surveys in prevalence of 1–2 and ≥ 3 limitations was examined using a weighted multinomial model, separately for ADL and IADL. The treatment of age and covariates were the same as in the main analyses.

Analyses were conducted using Stata software version 15.1 (StataCorp, 2017, College Station, TX: StataCorp LLC) and the figures were plotted using R software (R Core Team, Vienna, Austria, 2021). Estimates were reported with 95% confidence intervals (95%CI) and two-tailed p-values considered significant at 0.05 level. All methods were performed in accordance with the relevant guidelines and regulations.

### Statement of ethics

Informed consent was obtained from all subjects or their legal guardian(s). The surveys were declared to be of public interest by the national council for statistical information (Conseil National d’Information Statistique) and were approved by French Data Protection Authority (Commission Nationale de l’Informatique et des Libertés, French law no. 78-17).

## Results

Data on ADL and IADL were available on 13,584 participants in HS-2008 and 13,044 participants in CARE-2015, flowchart shown in Fig. S1. The mean age of participants was 72.0 (SD = 8.5) and 72.3 (SD = 8.8) years, 56.8% (N = 8351) and 55.9% (N = 8280) were women, and 3.3% (N = 3738) and 3.2% (N = 2623) lived in institutions in HS-2008 and CARE-2015 respectively. Table 1 presents the characteristics of men and women of both surveys.

**Table 1:** Characteristics of participants in the HS-2008 and CARE-2015 surveys.

		HS-2008 (n = 13,584)		CARE-2015 (n = 13,044)	
		Men (n = 5233)	Women (n = 8351)	Men (n = 4764)	Women (n = 8280)
Age	Mean (SD)	70.9 (8.1)	72.8 (8.7)	71.4 (8.3)	73.0 (9.2)
	(95% CI)	(70.6, 71.3)	(72.5, 73.2)	(71.0, 71.7)	(72.7, 73.3)
Resident in an institution	n	1236	2502	694	1929
	% (95% CI)	2.2 (1.9, 2.4)	4.1 (3.9, 4.4)	1.9 (1.8, 2.1)	4.2 (3.9, 4.5)
**Education**					
None	n	2048	3485	1167	2452
	% (95% CI)	24.7 (22.3, 26.6)	28.4 (26.7, 30.0)	16.1 (14.6, 17.7)	19.1 (17.7, 20.6)
Primary	n	1531	3250	1204	2918
	% (95% CI)	30.5 (28.4, 32.7)	42.5 (40.5, 44.4)	21.1 (19.3, 22.9)	27.8 (26.1, 29.5)
High school	n	897	802	1358	1657
	% (95% CI)	24.1 (22.0, 26.2)	14.5 (13.0, 16.0)	32.5 (30.3, 34.6)	28.6 (26.8, 30.5)
Baccalaureate	n	243	400	412	655
	% (95% CI)	5.8 (4.6, 7.0)	6.8 (5.7, 7.8)	11.4 (9.9, 12.9)	11.1 (9.8, 12.5)
University degree	n	514	414	623	598
	% (95% CI)	14.9 (13.1, 16.7)	7.9 (6.8, 9.1)	18.4 (17.0, 20.8)	13.3 (11.9, 14.8)
**Chronic conditions**					
Arthritis	n	1851	4074	2241	5207
	% (95% CI)	32.6 (30.4, 34.7)	46.6 (44.7, 48.6)	39.2 (37.0, 41.4)	56.6 (54.6, 58.6)
Cancer	n	596	730	415	504
	% (95% CI)	9.6 (8.3, 10.9)	9.1 (8.1, 10.2)	5.5 (4.7, 6.4)	5.5 (4.8, 6.3)
Heart disease	n	1189	1434	1191	1488
	% (95% CI)	19.0 (17.4, 20.7)	11.2 (10.2, 12.2)	15.8 (14.3, 17.3)	10.2 (9.3, 11.2)
Neurodegenerative disease	n	506	1158	505	1252
	% (95% CI)	3.1 (2.6, 3.6)	4.0 (3.5, 4.5)	2.4 (2.0, 2.8)	3.9 (3.5, 4.3)
Depression	n	344	1077	506	1394
	% (95% CI)	3.4 (2.6, 4.1)	6.9 (6.2, 7.7)	4.1 (3.4, 4.7)	9.2 (8.3, 10.2)
Diabetes	n	975	1322	981	1342
	% (95% CI)	15.0 (13.5, 16.6)	10.8 (9.7, 11.9)	16.1 (14.5, 17.7)	11.5 (10.4, 12.6)
Musculoskeletal disorders	n	1167	2135	2002	4114
	% (95% CI)	23.4 (21.5, 25.4)	27.1 (25.4, 28.8)	35.3 (33.1, 37.4)	43.1 (41.2, 45.0)
Stroke	n	549	730	415	504
	% (95% CI)	5.2 (4.3, 6.0)	3.8 (3.2, 4.4)	3.5 (2.9, 4.1)	2.8 (2.3, 3.3)
**Multimorbidity**^**a**^					
< 2 Chronic conditions	n	3151	4541	2241	3192
	% (95% CI)	68.7 (66.7, 70.8)	66.3 (64.6, 68.0)	63.0 (60.9, 65.1)	54.6 (52.7, 56.5)
≥ 2 Chronic conditions	n	2082	3810	2523	5088
	% (95% CI)	31.3 (29.2, 33.3)	3.7 (31.9, 35.4)	37.0 (34.9, 39.1)	45.4 (43.5, 47.3)
**ADL limitations**					
0	n	3765	5148	3480	5160
	% (95% CI)	92.3 (91.5, 93.2)	87.4 (86.5, 88.2)	92.8 (92.0, 93.6)	88.6 (87.8, 89.4)
1–2	n	643	1395	614	1370
	% (95% CI)	4.7 (4.0, 5.4)	8.0 (7.3, 8.8)	4.4 (3.8, 5.1)	6.6 (5.9, 7.3)
≥ 3	n	825	1808	670	1750
	% (95% CI)	3.0 (2.6, 3.4)	4.6 (4.2, 5.0)	2.8 (2.4, 3.2)	4.8 (4.4, 5.2)
**IADL limitations**					
0	n	3127	3726	2550	2947
	% (95% CI)	86.7 (85.6, 87.9)	76.2 (74.9, 77.5)	84.2 (83.6, 86.0)	74.8 (73.4, 76.1)
1–2	n	687	1474	739	1514
	% (95% CI)	5.8 (4.9, 6.6)	11.6 (10.6, 12.7)	8.1 (7.1, 9.2)	11.7 (10.7, 12.7)
≥ 3	n	1419	3151	1475	3819
	% (95% CI)	7.5 (6.6, 8.3)	12.2 (11.4, 13.0)	7.1 (6.4, 7.7)	13.5 (12.7, 14.4)

M, mean; SD, standard deviation; HS: Handicap Santé; CARE: Capacities, Aids and REssources; ADL: Activities of Daily Living; IADL: Instrumental Activities of Daily Living.^a^Using the chronic conditions listed above.

Data are n, followed by weighted % and 95% CI, unless stated otherwise.

**Figure 1:**
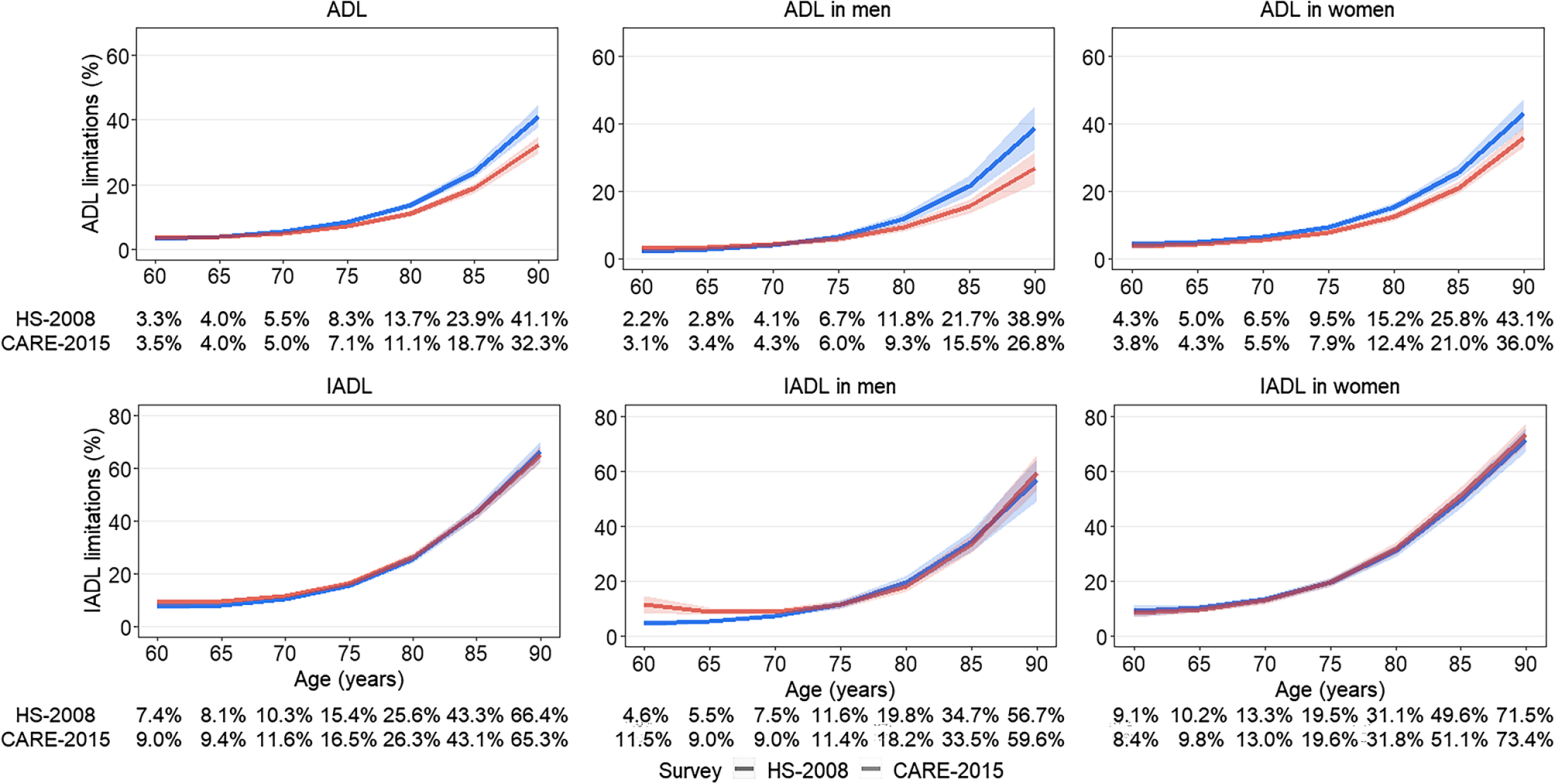
Prevalence of ADL and IADL limitations in HS-2008 and CARE-2015 surveys^a^ and by sex^b^. HS: Handicap Santé; CARE: Capacities, Aids and REssources; ADL: Activities of Daily Living; IADL: Instrumental Activities of Daily Living.^a^Estimated using weighted logistic models adjusted for survey (HS-2008, CARE-2015), age, age^2^, survey x age, sex, multimorbidity, education, and for significant interactions between covariates and age terms.^b^Estimated using weighted logistic models stratified on sex and adjusted for survey (HS-2008, CARE-2015), age, age^2^, survey x age, multimorbidity, education and for significant interactions between covariates and age terms.

The prevalence of ADL limitations was 10.5% and 9.6% in the 2008 and 2015 surveys respectively; Fig. 1 (top left panel) shows the weighted prevalence of ADL limitations in HS-2008 and CARE-2015 as a function of age. Prevalence of ADL increased with age, from 3.3% at age 60 to 41.1% at age 90 in HS-2008 and from 3.5% to 32.3% at similar ages in CARE-2015. Formal comparisons of change in prevalence between the surveys (Table 2) showed ADL limitations to have decreased at the 2015 survey, starting at age 75 (− 1.2%, 95% CI − 2.0, − 0.4%; p-value = 0.003) and continuing to age 90 (− 8.8%, 95% CI − 12.7, − 5.0%; p-value < 0.001). This decrease was larger at older ages (p-value for survey by age interaction = 0.01).

**Table 2:** Difference between HS-2008 and CARE-2015 in ADL and IADL limitations as a function of age and by sex.

Age (years)	≥ 1 ADL limitation					
	Overall		Men		Women	
	Difference in prevalence^a,c^ (95% CI)	p	Difference in prevalence^b,^^c^ (95% CI)	p	Difference in prevalence ^b,^^c^ (95% CI)	p
60	0.2 (− 0.6, 1.0)	0.67	0.9 (− 0.2, 2.0)	0.10	-0.4 (− 1.6, 0.8)	0.47
65	0.1 (− 0.8, 0.7)	0.85	0.6 (− 0.3, 1.6)	0.20	− 0.6 (− 1.7, 0.4)	0.24
70	− 0.5 (− 1.2, 0.3)	0.20	0.2 (− 0.8, 1.1)	0.70	− 1.0 (− 2.1, 0.1)	0.06
75	− 1.2 (− 2.0, − 0.4)	0.003	− 0.7 (− 1.7, 0.4)	0.23	− 1.7 (− 2.8, − 0.5)	0.004
80	− 2.6 (− 3.7, − 1.5)	< 0.001	− 2.5 (− 4.2, − 0.8)	0.005	− 2.8 (− 4.4, − 1.3)	< 0.001
85	− 5.2 (− 7.3, − 3.1)	< 0.001	− 6.2 (− 9.7, − 2.7)	< 0.001	− 4.8 (− 7.5, − 2.1)	< 0.001
90	− 8.8 (− 12.7, − 5.0)	< 0.001	− 12.1 (− 18.7, − 5.5)	< 0.001	− 7.1 (− 11.8, − 2.4)	0.003
P for trend^d^	0.01		0.003		0.38	

Age (years)	≥ 1 IADL limitation					
	Overall		Men		Women	
	Difference in prevalence ^a,c^ (95% CI)	p	Difference in prevalence ^b,^^c^ (95% CI)	p	Difference in prevalence ^b,^^c^ (95% CI)	p
60	1.6 (0.1, 3.2)	0.04	7.0 (3.6, 10.4)	< 0.001	− 0.6 (− 2.8, 1.5)	0.56
65	1.4 (0.1, 2.6)	0.04	3.5 (1.9, 5.2)	< 0.001	− 0.5 (− 2.4, 1.4)	0.63
70	1.2 (0.1, 2.4)	0.04	1.5 (− 0.2, 3.1)	0.08	− 0.3 (− 2.1, 1.5)	0.78
75	1.1 (− 0.2, 2.4)	0.10	− 0.2 (− 2.4, 2.0)	0.86	0.1 (− 1.9, 2.1)	0.93
80	0.7 (− 1.2, 2.6)	0.45	− 1.5 (− 4.6, 1.5)	0.32	0.7 (− 2.0, 3.5)	0.60
85	− 0.1 (− 3.3, 3.0)	0.94	− 1.2 (− 6.1, 3.7)	0.64	1.6 (− 2.6, 5.7)	0.46
90	− 1.0 (− 4.8, 2.8)	0.59	− 2.9 (− 6.9, 12.7)	0.56	1.8 (− 2.6, 6.3)	0.42
P for trend^d^	0.12		< 0.001		0.43	

HS, Handicap Santé; CARE, Capacities, Aids and REssources; ADL, Activities of Daily Living; IADL, Instrumental Activities of Daily Living.

^a^Estimated using weighted logistic models adjusted for survey (HS-2008, CARE-2015), age, age^2^, survey × age, sex, multimorbidity, education, and for significant interactions between covariates and age terms.

^b^Estimated using weighted logistic models stratified on sex and adjusted for survey (HS-2008, CARE-2015), age, age^2^, survey × age, multimorbidity, education and for significant interactions between covariates and age terms.

^c^Negative values indicate higher prevalence in HS-2008 compared to CARE-2015.

^d^p value for survey by age interaction (Wald test) to test whether differences by surveys vary by age.

The overall prevalence of IADL limitations was 19.3% in HS-2008 and 20.8% in CARE-2015. Figure 1 (bottom left panel) shows higher rates at older ages, from 7.4% and 9.0% in HS-2008 and CARE-2015 respectively at age 60 to 66.4% and 65.3% at age 90. IADL prevalence was somewhat higher in 2015 compared to 2008 (Table 2) from age 60 (1.6, 95% CI 0.1, 3.2; p-value = 0.04) to age 70 (1.2, 95% CI 0.1, 2.4; p-value = 0.04) but not beyond this age. Overall change in IADL limitations between the two surveys did not differ by age (p-value for survey by age interaction = 0.12).

ADL and IADL rates were generally higher in women compared to men in both surveys (Fig. 1) and the age-related trends in change in limitations between the two surveys differed by sex in both ADL (p-value for interaction between survey x sex x age = 0.05) and IADL (p-value for interaction between survey × sex × age = 0.01). Although the prevalence of ADL declined in both men and women (Table 2), the decline was somewhat larger in the oldest age-groups in men. Sex differences in change in IADL was due to an increase in limitations in men from age 60 (7.0, 95% CI 3.6, 10.4; p-value = 0.001) to age 65 (3.5, 95% CI 1.9, 5.2; p-value < 0.001), no change in IADL limitations were observed in women (Table 2).

**Figure 2:**
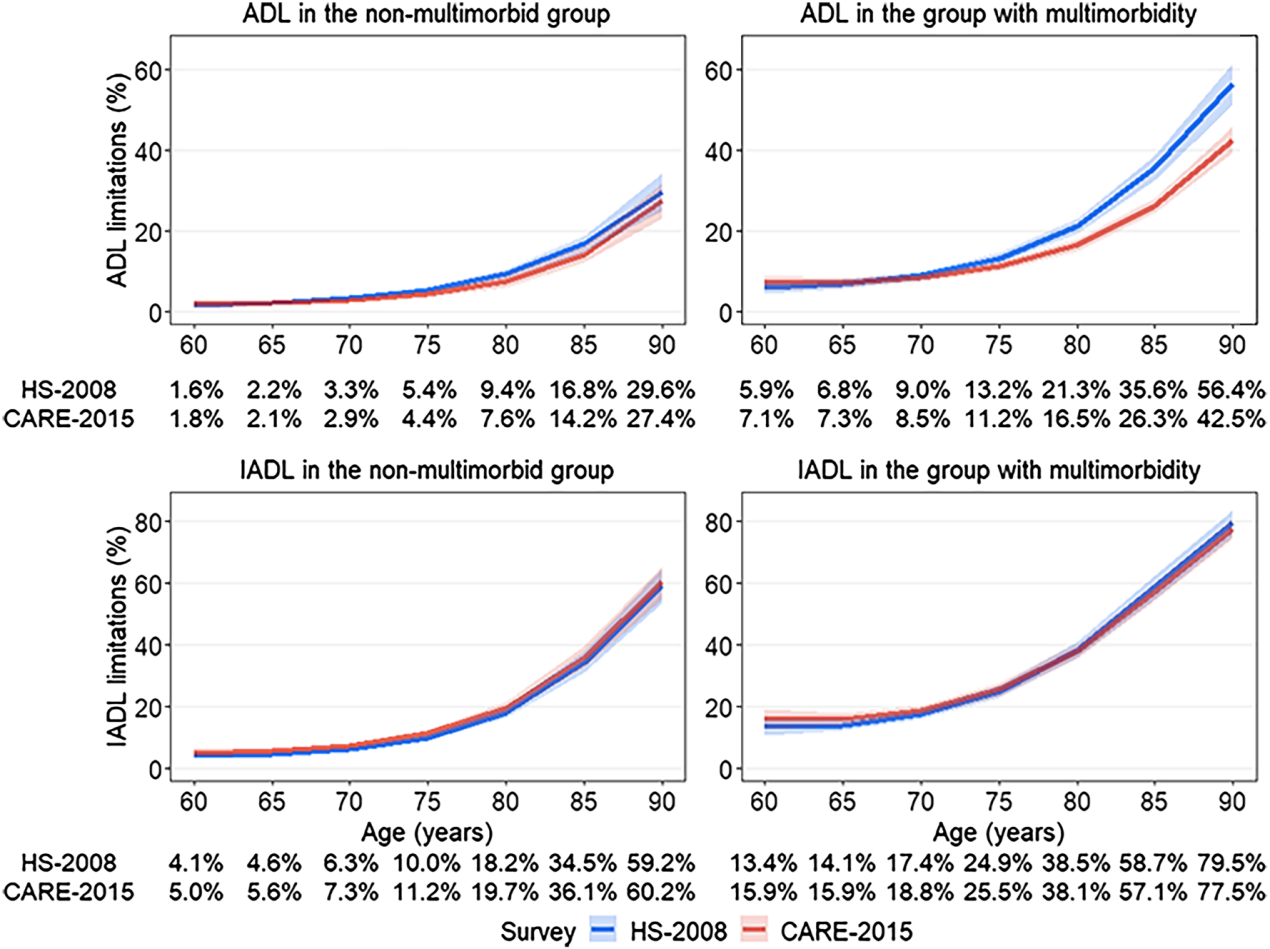
Prevalence of ADL and IADL limitations in HS-2008 and CARE-20155 survey, by age and multimorbidity status^a^ . HS: Handicap Santé,;CARE: Capacities, Aids and REssources; ADL: Activities of Daily Living; IADL: Instrumental Activities of Daily Living.^a^Estimated using weighted logistic models stratified on multimorbidity status and adjusted for survey (HS-2008, CARE-2015), age, age^2^, survey x age, sex, education, and for significant interactions between covariates and age terms.

Multimorbidity was reported by 37.4% of the population across the two surveys, it was higher in CARE-2015 than HS-2008 (41.6% vs 32.6%; p-value < 0.001), and in women compared to men (39.7% vs 34.3%; p-value < 0.001). Participants with multimorbidity had higher rates of ADL (18.1% vs 6.8% in HS-2008 and 15.5% vs 5.4% in CARE-2015, both p-value < 0.001) and IADL limitations (31.8% vs 13.2% in HS-2008 and 32.1% vs 12.8% in CARE-2015, both p-value < 0.001), Table S1. In the overall population, prevalence of ADL, adjusted for age and sex, was 6.1% for participants without multimorbidity and 16.6% for those with multimorbidity, they were respectively 13.0% and 31.9% for IADL, Table S2. There was some evidence of difference in change in ADL limitations as a function of multimorbidity status (p-value for interaction between survey, multimorbidity, and age = 0.06). In people with multimorbidity, the prevalence of ADL declined, particularly at older ages (p-value for survey by age interaction = 0.001; Fig. 2, top right panel) from age 75 (-2%, 95% CI: − 3.6, − 0.4%; p-value = 0.01) to age 90 (− 13.9, 95% CI: − 19.3, − 8.4%; p-value < 0.001), Table S3. In individuals free of multimorbidity, there was no change in ADL limitations between the two surveys (Table S3). IADL limitations did not change as a function of multimorbidity (p-value for interaction for survey by multimorbidity = 0.83 and for survey by multimorbidity by age = 0.35; Fig. 2 bottom panel, and Table S3).

**Figure 3:**
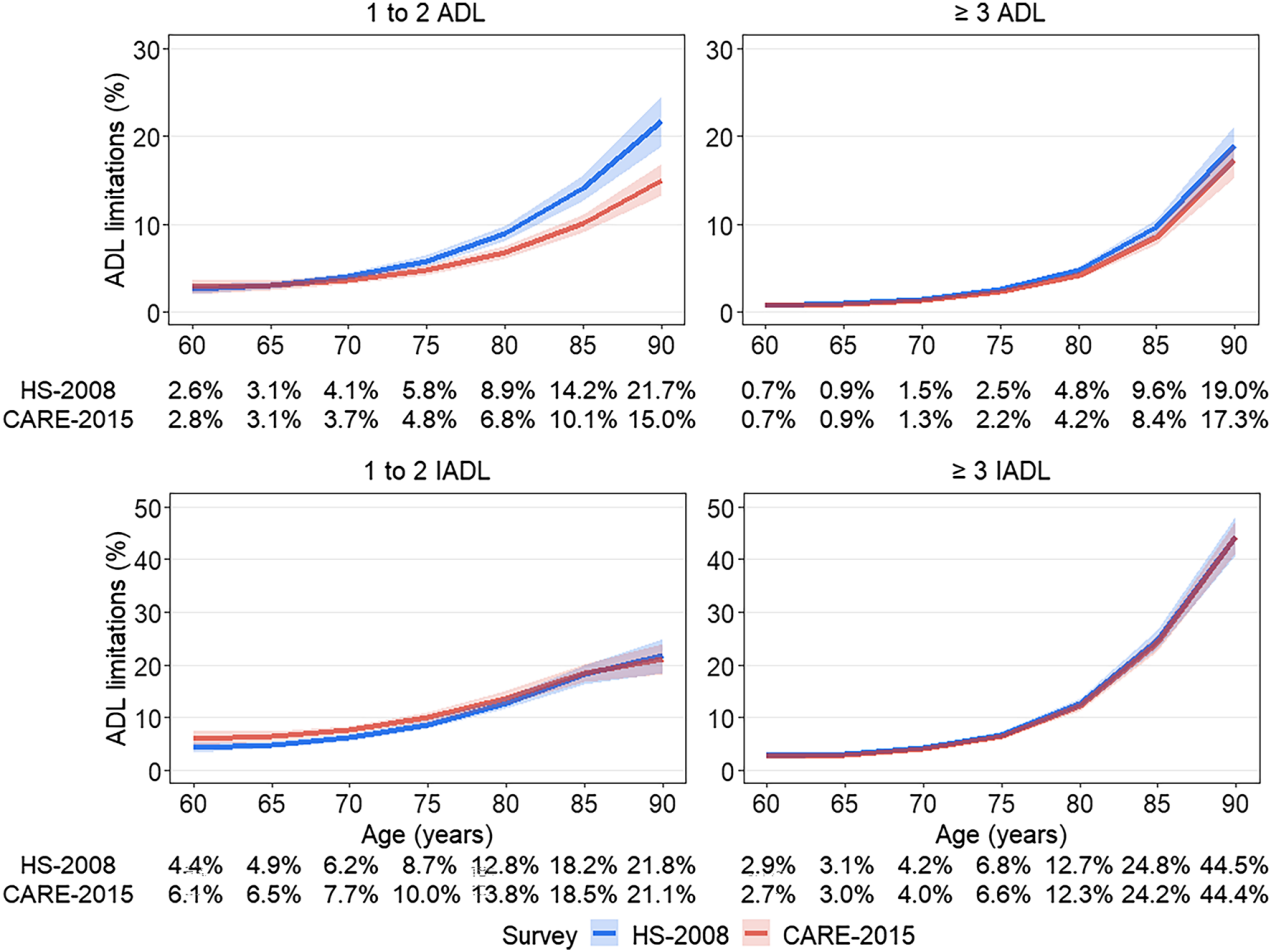
Prevalence of ADL and IADL limitations in HS-2008 and CARE-2015 survey, by age and number of reported limitations^a^ . HS: Handicap Santé,;CARE: Capacities, Aids and REssources; ADL: Activities of Daily Living; IADL: Instrumental Activities of Daily Living.^a^Estimated using weighted multinomial models for 1–2 and ≥ 3 limitations, and adjusted for survey (HS-2008, CARE-2015), age, age^2^, survey x age, sex, multimorbidity, education, and for significant interactions between covariates and age terms.

The prevalence of participants with one to two ADL limitations increased from 2.6% at age 60 to 21.7% at age 90 in HS-2008 and from 2.8 to 15.0% in CARE-2015 at similar ages (Fig. 3, top left panel). At age 60, three or more ADL limitations was reported by 0.7% in both surveys, increasing at age 90 to 19.0% in HS-2008 and 17.3% in CARE-2015 (Fig. 3, top right panel). The prevalence of one to two ADL limitations (Table S4) decreased in 2015 compared to 2008, from age 75 (− 1.0%; 95% CI: − 1.7, − 0.3%; p-value = 0.005) to age 90 years (− 6.7%; 95% CI: − 9.9, − 3.6%; p-value < 0.001). A small decrease in prevalence of ≥ 3 ADL limitations was observed at age 80 (− 0.6%; 95% CI: − 1.1, − 0.1%; p-value = 0.02) and age 85 (− 1.2%; 95% CI: − 2.2, − 0.1%; p-value = 0.03).

The prevalence of one to two IADL limitations increased from 4.4% and 6.1% at age 60 to 21.8% and 21.1% at age 90 in HS-2008 and CARE-2015 respectively (Fig. 3, bottom left panel). The corresponding numbers for three or more IADL limitations were 2.9% and 2.7% at age 60 and 44.5% and 44.4% at age 90 (Fig. 3, bottom right panel). The analysis of change in prevalence of one to two IADL limitations showed from age 60 (1.8%; 95% CI: 0.4, 3.2%; p-value < 0.001) to age 75 (1.3%; 95% CI: 0.2, 2.4%; p-value < 0.001) but not at older ages, Table S4. There was no change in ≥ 3 IADL limitations between the surveys.

## Discussion

This study of changes in functional limitations, based on two nationally representative surveys conducted 7 years apart in France, presents four key findings. One, a temporal trend of decline in ADL limitations between 2008 and 2015 was observed, this was particularly true among adults older than 75 years with the largest improvements seen at age 90. Two, further analyses aimed at examining severity of limitations shows that decline in ADL was evident in those with one to 2 limitations but less pronounced in those with three of more limitations. Three, decline in ADL was primarily seen in those with multimorbidity. Four, there was some evidence of an increase in IADL limitations in men aged 60 to 70 years but not in women or in men beyond 70 years.

A trend of decline in ADL limitations, starting in the 1980s, has been shown in various population settings whereas results on change in IADL limitations are inconsistent^5,9–13^. We extend these findings by including people beyond the 8th decade of life, where the improvements in ADL were the largest. Much of the research on ADL and IADL uses a dichotomous measure, with 1 or more limitation grouped together such that few previous studies have considered trends for severity of limitations. One study, using data from Cognitive Function and Ageing Studies, compared participants with severe disability (unable to undertake at least one of five ADL activities without human help), mild disability (able to undertake ADL activities but need help for at least one of of two IADL) and participants free of disability. They reported less severe disability to be increasing but more severe disability to be unchanged^27^. Another study used the number of reported limitations in data spanning 2004 to 2014 to show no differences in trend with severity levels^13^.

In the present study the conventional definition of functional limitation was used, whereby functional limitations refer to major difficulties or the complete inability to perform daily tasks. Using this definition, severity defined as the number of limitations, showed a compression of ADL when up to two limitations were considered but the results were less pronounced for three or more limitations. The improvement in ADL limitations was highest at older ages, as also seen in previous studies from the United States in data up to 2008 where one or more limitations were used to define ADL disability and severity of limitations was not considered^10,33^. Our data seem not to support the dynamic equilibrium hypothesis^34^ that predicts increase in life expectancy to be accompanied by increased prevalence of modest disability but a decrease in severe disability. Our data suggest compression of disability when defined as up to two ADL limitations but not the 3 or more ADL limitations definition, a concern as 3.9% of the French population reported 3 or more limitations.

Besides the general trend in declining ADL limitations we also found an increase in IADL rates, particularly in men aged 60 to 70 years. The extent to which this finding reflects a period effect or is specific to men in France remains unclear and needs further investigation. Changes occurring between 2008 and 2015 (the 2010 pension reform) or other factors that affect survival of participants according to their birth cohort (World War II) may have contributed to these observed differences. Data from the two surveys included in the analyses do not allow further insight into these findings but surveys in the future in France and similar surveys in other high-income countries would provide an explanation for these findings.

The prevalence of chronic diseases and multimorbidity has increased worldwide^35^; in our study multimorbidity in France was higher in 2015 than in 2008. Chronic diseases are major contributors to functional limitations, and there is emerging evidence that their impact on functional limitations has declined in recent years^36–38^. However, the single disease approach does not address the fact that over 50% of adults aged 65 years and older have two or more chronic diseases^3^. To our knowledge, no previous study has examined temporal changes in ADL and IADL as a function of multimorbidity. We observed an increase in prevalence of multimorbidity, driven mainly by an increase in the prevalence of musculoskeletal and rheumatological chronic conditions. Consistent with studies on individual chronic diseases^36–38^, we found a greater decline in the prevalence of ADL limitations in participants with multimorbidity. Better management of major chronic diseases is likely to have contributed to reducing the impact of multimorbidity on functional limitations. It is also possible that the oldest age-groups have better access to services and assistive technologies^36–38^.

Sex differences in ADL or IADL prevalence have been examined in several studies with women consistently reporting more limitations than men^39,40^, but results on trends over time remain inconsistent. Some studies show larger improvement in functional limitations in women^14,15^ or similar trends in men and women^16,17^ but these patterns have rarely been examined as a function of the age of participants. In the present study, the prevalence of ADL or IADL limitations and multimorbidity was higher in women, and change in limitations were not similar in men and women. The most notable difference was observed in IADL limitations where men but not women aged 60–65 showed an increase in 2015 compared to 2008. The 7-year period used in this study is admittedly not sufficient to reflect the impact of the dynamics of sex/gender-related structural changes.

Findings from the present study need to be considered in light of its strengths and limitations. The primary strength is the use of weighted national surveys with a similar data collection strategy at both surveys in 2008 and 2015. The explicit effort to collect data on a nationally representative sample, with inclusion of the oldest age groups and people living in institutions is a further strength as these groups are often overlooked groups in studies on functional limitations. The oversampling of people registered as being disabled ensures that this group is also represented in the analyses of changes in ADL and IADL limitations.

This study also has a number of limitations. First, unavoidable differences in data collection between the two surveys may partially explain the findings despite both surveys being similar in design and equally representative of the French population. This is particularly the case for reported chronic diseases where the prevalence may be influenced by contextual factors. Second, the measure of multimorbidity in the surveys was not optimal as it was based on self-reported chronic conditions. Further, a restricted list of chronic conditions was used and it may not fully reflect the conditions experienced at older ages. Third, the disentanglement of temporality, along with age and birth cohort effects requires more comprehensive data. Repeated cross-sectional analysis shed light on the health of the population and its change but cannot be interpreted as individual trajectories nor allow conclusions to be drawn on the efficacy of public health policies. Fourth, the assessment of functional limitations was based on the number of reported limitations which is the most common approach^41^ but it is only a partial reflection of the complexity of disability. There is little consensus on how best to define severity of ADL and IADL limitations, making it difficult to compare findings across studies.

The critical question that needs to be addressed by future studies is to understand the divergence in trends for IADL and ADL. While ADL reflects disability, the IADL measures better reflect the ability to live independently and function without the need for assistance. Public health policies promoting access to services and assistive technologies may have contributed to a decline in ADL limitations^36–38^, particularly those with the least severe impairments where these interventions are readily managed. The public policy implications of our findings concern 1) the lack of improvement in those with severe ADL limitations and 2) the lack of improvement in IADL disability. The second of these concerns is particularly salient in the context of sociodemographic changes implying that older adults are increasingly likely to live alone, without immediate family in the vicinity, and that rapid changes in the technological sphere imply that most day to day activities require a certain level of cognitive autonomy.

In conclusion, these nationally representative data show that despite an extension of multimorbidity the prevalence of ADL limitations has decreased in France between 2008 to 2015. This was particularly the case in the oldest age-group, in the tenth decade of life. The lack of improvement in IADL highlights the need to uncover the barriers for improvement in cognitively challenging instrumental activities. These findings are likely to apply to high-income countries with universal health care. Whether similar improvements in ADL are also seen in middle- and low-income countries remains unclear.

## Supplementary Information

Supplementary Information.

## Data Availability

The data that support the findings of this study are available from INSEE portal (HS-2018 “http://www.progedo-adisp.fr/enquetes/XML/lil-0520.xml”; CARE-2015 “http://www.progedo-adisp.fr/enquetes/XML/lil.php?lil=lil-1236”) but restrictions apply to the availability of these data, which were used under license for the current study, and so are not publicly available. Data are however available from the corresponding author upon reasonable request and with permission of INSEE.
