# Supplementary material for: The role of age, sex, and multimorbidity in 7-year change in prevalence of limitations in adults 60–94 years
Source: Sci Rep. 2022 Oct 31;12:18270. doi: 10.1038/s41598-022-23053-8 (PMC9622834; doi:10.1038/s41598-022-23053-8)
Supplement: Supplementary file 1 — Supplementary Information. [file 41598_2022_23053_MOESM1_ESM.docx]

**Supplemental data**

**The role of age, sex, and multimorbidity in 7-year change in prevalence of limitations in adults 60 to 94 years**

Benjamin Landré^1^*, PhD, Andres Gil-Salcedo,^1^, MPH, Louis Jacob,^1,2,3^, MD, PhD, Alexis Schnitzler,^1^ MD, PhD, Aline Dugravot,^1^ MPH, Séverine Sabia,^1,4^¤ PhD, Archana Singh-Manoux,^1,4^¤ PhD

¤Equal contribution

^1^Université de Paris, Inserm U1153, Epidemiology of Ageing and Neurodegenerative diseases, France

^2^Research and Development Unit, Parc Sanitari Sant Joan de Déu, CIBERSAM, Sant Boi de Llobregat, Barcelona, Spain

^3^Faculty of Medicine, University of Versailles Saint-Quentin-en-Yvelines, Montigny-le-Bretonneux, France

^4^Department of Epidemiology and Public Health, University College London, UK

*CORRESPONDING AUTHOR

Benjamin Landré

Université Paris Cité, Inserm U1153

EpiAgeing “Epidemiology of Ageing & Neurodegenerative diseases”

10 avenue de Verdun, Paris 75010, France

Tel : +33 (0)1 57 27 90 46

Email: [benjamin.landre@inserm.fr](mailto:benjamin.landre@inserm.fr)

ORCID: 0000-0002-3893-4197

**Figure S1: flow chart.**

**
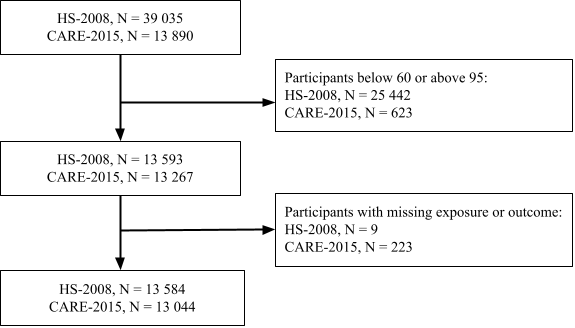
**

**Table S1: Characteristics of participants as a function of multimorbidity.^a^**

|  |  | **HS-2008**  (n = 13584) | |  | **CARE-2015**  (n = 13044) | |
| --- | --- | --- | --- | --- | --- | --- |
|  |  | Multimorbid  (n= 5892) | Non- multimorbid  (n= 7692) |  | Multimorbid  (n= 7611) | Non- multimorbid  (n= 5433) |
| **Age** | Mean (SD) | 73.9 (8.4) | 71.1 (8.4) |  | 74.3 (9.1) | 70.9 (8.4) |
| **Sex** | Women | 3810 (58.6) | 4541 (56.0) |  | 5088 (60.8) | 3192 (52.3) |
| **Resident in an institution** | | 1527 (4.6) | 2211 (2.7) |  | 1436 (4.1) | 1187 (2.5) |
| **Education** | None | 2414 (30.2) | 3119 (25.1) |  | 2298 (22.5) | 1321 (14.4) |
|  | Primary | 2224 (40.0) | 2557 (36.0) |  | 2542 (28.6) | 1580 (22.1) |
|  | High school | 722 (17.0) | 977 (19.4) |  | 1715 (29.7) | 1300 (30.7) |
|  | Baccalaureate | 224 (4.9) | 419 (7.1) |  | 517 (8.6) | 550 (13.2) |
|  | University degree | 308 (7.9) | 620 (12.4) |  | 539 (10.5) | 682 (19.5) |
| **Chronic conditions** |  |  |  |  |  |  |
| Arthritis |  | 4118 (76.0) | 1807 (23.4) |  | 1208 (86.4) | 6240 (22.1) |
| Cancer |  | 1068 (19.1) | 371 (4.6) |  | 371 (10.2) | 858 (2.2) |
| Heart disease |  | 2125 (33.1) | 498 (5.6) |  | 260 (25.3) | 2419 (3.7) |
| Neurodegenerative disease | | 1119 (7.6) | 545 (1.7) |  | 491 (5.5) | 1266 (1.6) |
| Depression |  | 1176 (14.0) | 245 (1.2) |  | 149 (14.5) | 1751 (1.5) |
| Diabetes |  | 1748 (26.3) | 549 (6.0) |  | 331 (24.0) | 1992 (6.1) |
| Musculoskeletal disorders |  | 2737 (57.7) | 565 (10.0) |  | 567 (79.0) | 5549 (11.5) |
| Stroke |  | 998 (10.6) | 281 (1.4) |  | 130 (6.1) | 789 (1.0) |
| **ADL** | None | 3462 (81.9) | 5451 (93.2) |  | 4625 (84.6) | 4015 (94.6) |
|  | One or two | 1100 (11.5) | 938 (4.2) |  | 1416 (9.1) | 568 (3.2) |
|  | Three or more | 1330 (6.6) | 1303 (2.6) |  | 1570 (6.4) | 850 (2.2) |
| **IADL** | None | 2456 (68.2) | 4397 (86.8) |  | 2579 (67.9) | 2918 (87.3) |
|  | One or two | 1090 (14.5) | 1071 (6.5) |  | 1523 (15.1) | 730 (6.6) |
|  | Three or more | 2346 (17.3) | 2224 (6.7) |  | 3509 (17.0) | 1785 (6.2) |

Notes. Abbreviations: M, mean; SD, standard deviation; HS: Handicap Santé; CARE: Capacities, Aids and REssources; ADL: Activities of Daily Living; IADL: Instrumental Activities of Daily Living.
^a^Multimorbidity was defined as 2 or more of the listed chronic diseases.
Data are N (weighted %) unless stated otherwise.

**Table S2: Weighted prevalence of ADL and IADL limitations in the overall population by multimorbidity status.**

|  | **≥ 1 ADL limitation** | **≥ 1 IADL limitation** |
| --- | --- | --- |
|  | Weighted prevalence  (95% CI)^a^ | Weighted prevalence (95% CI)^a^ |
| No multimorbidity | 6.1% (5.7, 6.5) | 13.0% (12.3, 13.6) |
| Multimorbidity | 16.6% (15.8, 17.3) | 31.9% (30.9, 33.0) |

Notes. Abbreviations: ADL: Activities of Daily Living; IADL: Instrumental Activities of Daily Living.
^a^Estimated using weighted logistic models stratified on multimorbidity status and adjusted for age, age², and sex.

**Table S3: Difference between HS-2008 and CARE-2015 in ADL and IADL limitations by age and multimorbidity status.**

|  | **≥ 1 ADL limitation** | | | | |  | |
| --- | --- | --- | --- | --- | --- | --- | --- |
|  | **Multimorbidity** | | **No multimorbidity** | |  | |  |
| Age (years) | Difference  in prevalence ^a,b^  (95% CI) | P | Difference  in prevalence ^a,b^  (95% CI) | P |  | |  |
| 60 | 1.2 (-0.8, 3.1) | 0.24 | 0.2 (-0.9, 1.3) | 0.71 |  | |  |
| 65 | 0.4 (-1.2, 2.1) | 0.61 | -0.1 (-0.8, 0.7) | 0.92 |  | |  |
| 70 | -0.5 (-2.0, 1.0) | 0.53 | -0.5 (-1.2, 0.3) | 0.21 |  | |  |
| 75 | -2.0 (-3.6, -0.4) | 0.01 | -1.0 (-2.3, 0.4) | 0.10 |  | |  |
| 80 | -4.8 (-6.9, -2.6) | < 0.001 | -1.8 (-3.8, 0.1) | 0.07 |  | |  |
| 85 | -9.2 (-12.9, -5.6) | < 0.001 | -2.6 (-5.4, 0.3) | 0.08 |  | |  |
| 90 | -13.9 (-19.3, -8.4) | < 0.001 | -2.2 (-8.4, 4.0) | 0.49 |  | |  |
| P for trend^c^ | 0.001 |  | 0.37 |  |  | |  |
|  | **≥ 1 IADL limitation** | | | |  | |  |
|  | **Multimorbidity** | | **No Multimorbidity** | |  | |  |
| Age (years) | Difference  in prevalence ^a,b^  (95% CI) | P | Difference  in prevalence ^a,b^  (95% CI) | P |  | |  |
| 60 | 2.5 (-1.1, 6.0) | 0.17 | 0.9 (-0.5, 2.4) | 0.20 |  | |  |
| 65 | 1.8 (-0.9, 4.6) | 0.19 | 0.9 (-0.3, 2.2) | 0.15 |  | |  |
| 70 | 1.3 (-1.0, 3.7) | 0.27 | 1.0 (-0.2, 2.2) | 0.10 |  | |  |
| 75 | 0.7 (-1.8, 3.1) | 0.58 | 1.2 (-0.2, 2.7) | 0.10 |  | |  |
| 80 | -0.4 (-3.7, 3.0) | 0.83 | 1.6 (-0.9, 4.0) | 0.21 |  | |  |
| 85 | -1.6 (-6.2, 2.9) | 0.48 | 1.6 (-2.9, 6.2) | 0.48 |  | |  |
| 90 | -2.0 (-6.2, 2.2) | 0.34 | 1.0 (-5.1, 7.2) | 0.74 |  | |  |
| P for trend^c^ | 0.19 |  | 0.51 |  |  | |  |

Notes. Abbreviations: HS: Handicap Santé; CARE: Capacities, Aids and REssources; ADL: Activities of Daily Living; IADL: Instrumental Activities of Daily Living.
^a^Estimated using weighted logistic models stratified on multimorbidity status and adjusted for survey (HS-2008, CARE-2015), age, age², survey x age, sex, education, and for significant interactions between covariates and age terms.
^b^Negative values indicate higher prevalence of limitations in HS-2008.

^c^P value for survey by age interaction (Wald test) to test whether differences by surveys vary by age.

**Table S4: Difference between HS-2008 and CARE-2015 in the number of ADL and IADL limitations by age.**

|  | **ADL** | | | | |  | |
| --- | --- | --- | --- | --- | --- | --- | --- |
|  | **1 to 2 limitations** | | | **≥ 3 limitations** | |  | |
| Age (years) | Difference in prevalence ^a,b^  (95% CI) | P | Difference in prevalence ^a,b^  (95% CI) | | P | |  |
| 60 | 0.3 (-0.5, 1.1) | 0.51 | -0.0 (-0.2, 0.2) | | 0.85 | |  |
| 65 | -0.0 (-0.7, 0.7) | 0.99 | -0.1 (-0.3, 0.2) | | 0.61 | |  |
| 70 | -0.4 (-1.0, 0.3) | 0.27 | -0.1 (-0.4, 0.1) | | 0.31 | |  |
| 75 | -1.0 (-1.7, -0.3) | 0.005 | -0.3 (-0.6, 0.0) | | 0.08 | |  |
| 80 | -2.1 (-3.1, -1.2) | < 0.001 | -0.6 (-1.1, -0.1) | | 0.02 | |  |
| 85 | -4.1 (-5.8, -2.3) | < 0.001 | -1.2 (-2.2, -0.1) | | 0.03 | |  |
| 90 | -6.7 (-9.9, -3.6) | < 0.001 | -1.7 (-4.2, 0.7) | | 0.17 | |  |
| P for trend**^c^** | 0.006 |  | 0.30 | |  | |  |
|  | **IADL** | | | | | |  |
|  | **1 to 2 limitations** | | **≥ 3 limitations** | | | |  |
| Age (years) | Difference in prevalence ^a,b^  (95% CI) | P | Difference in prevalence ^a,b^  (95% CI) | | P | |  |
| 60 | 1.8 (0.4, 3.2) | 0.01 | -0.2 (-0.8, 0.5) | | 0.65 | |  |
| 65 | 1.6 (0.4, 2.7) | 0.008 | -0.2 (-0.7, 0.4) | | 0.59 | |  |
| 70 | 1.4 (0.4, 2.5) | 0.007 | -0.2 (-0.8, 0.4) | | 0.49 | |  |
| 75 | 1.3 (0.2, 2.4) | 0.02 | -0.3 (-1.0, 0.4) | | 0.40 | |  |
| 80 | 1.0 (-0.5, 2.5) | 0.20 | -0.5 (-1.6, 0.7) | | 0.42 | |  |
| 85 | 0.3 (-2.1, 2.7) | 0.79 | -0.6 (-2.9, 1.7) | | 0.63 | |  |
| 90 | -0.7 (-4.2, 2.8) | 0.69 | -0.1 (-4.1, 3.9) | | 0.96 | |  |
| P for trend**^c^** | 0.06 |  | 0.96 | |  | |  |

Notes. Abbreviations: HS: Handicap Santé; CARE: Capacities, Aids and REssources; ADL: Activities of Daily Living; IADL: Instrumental Activities of Daily Living.
^a^Estimated using weighted multinomial models for 1-2 and ≥3 limitations, and adjusted for survey (HS-2008, CARE-2015), age, age², survey x age, sex, multimorbidity, education, and for significant interactions between covariates and age terms.
^b^Negative values indicate higher prevalence of limitations in HS-2008.

^c^P value for survey by age interaction (Wald test) to test whether differences by surveys vary by age.
